# Proportion of Variant Anatomy of the Circle of Willis and Association with Vascular Anomalies on Cerebral CT Angiography

**DOI:** 10.1155/2019/6380801

**Published:** 2019-06-16

**Authors:** Roy Munialo Machasio, Rose Nyabanda, Timothy Musila Mutala

**Affiliations:** ^1^University of Nairobi, Department of Diagnostic Imaging and Radiation Medicine, Kenya; ^2^Kenyatta National Hospital, Kenya

## Abstract

**Background and Purpose:**

There is a wide variation in the anatomy of the CW in different individuals and population groups. The purpose of this study was to determine the proportion of variant anatomy of the circle of Willis (CW) and associated anomalies in patients with suspected cerebrovascular disease referred for cerebral computed tomography angiography (CTA) in two tertiary hospitals in Kenya.

**Methodology:**

This was a cross-sectional descriptive study conducted on 94 patients referred for cerebral CTA at the Kenyatta and Nairobi hospitals from August 2017 to February 2018. MIP and 3D reformatted images were analyzed by two senior radiologists to determine the final configuration of the CW and presence of vascular pathology. Vessels with diameters <0.8 mm were considered to be absent or hypoplastic. Chen et al. classification was used to determine the final configuration of CW.

**Results:**

Complete CW was seen in 37.2% (37.7% vs. 36.6% in males and females, respectively, p=0.909). Type A variant was the commonest anterior variant at 78.7%. Type E variant was the dominant posterior variant at 41.5%. Fetal PCA was demonstrated in 25.5%, unilateral > bilateral fetal PCA. Aneurysms were seen in 24.5% of patients. ACoA aneurysms were commonest at 43.6%. AVMs were seen in 8.5% of patients. Azygous ACA, fenestration, and duplication of vessels and persistent TA were not demonstrated. There is no significant association between aneurysms/AVMs and CW configuration.

**Conclusion:**

The findings in this study demonstrate slight differences in the CW configuration. A higher proportion of complete anterior CW was seen in female patients. Type A anterior and type E posterior circulation variants were the commonest in both males and females. No significant association was demonstrated between CW configuration and occurrence of aneurysms/AVMs in this study. Various methods of classification of CW configuration have been proposed with no standardized method to date, thus the need for consensus building between neuroanatomists, neurologists, neurosurgeons, and neuroradiologists for ease of comparison between similar future studies on the CW.

## 1. Introduction

The circle of Willis (CW) is an important anastomotic arterial polygon at the base of the brain that connects the carotid and vertebrobasilar systems [1–3]. It is an essential route for collateral supply of blood to the brain in cases of occlusion in either system [4, 5]. CW variants include fenestration, duplication, hypoplasia, or agenesis [6]. These variants influence the cerebral blood flow hemodynamics with influence on vascular territories, pathophysiology of cerebral arterial remodeling, aneurysm formation/rupture, and stroke development [7–11]. A complete CW configuration has also been associated with reduced risk of intracranial hemorrhage following intravenous thrombolysis in patients with ischaemic stroke [12]. Persistent embryonic carotid-vertebrobasilar anastomosis such as hypoglossal, proatlantal, otic, and persistent trigeminal arteries are extremely rare occurring in about 0.1%-0.6% of the general population [13]. CT angiography (CTA) is a fast reliable noninvasive method for evaluation of the circle of Willis as well as other intracranial arteries and aneurysms with high sensitivity and specificity comparable to gold standard catheter angiography [14].

Various methods have been proposed for classification of the morphology of the CW due to complex anterior and posterior circulation variations [1, 15–17]. Chen et al. classified variations in both anterior and posterior CW from A-J [18, 19]. For the purposes of this study, Chen et al. classification was used due to its simplicity as compared to the other methods.

The goal of the current study was to establish the proportion of variant anatomy of the CW and associated anomalies in patients with suspected cerebrovascular disease referred for cerebral CTA in two tertiary hospitals in Kenya.

## 2. Materials and Methods

This was a cross-sectional descriptive study carried out on patients referred for cerebral CTA at Kenyatta National Hospital and Nairobi Hospital, both located in Nairobi County, Kenya.

CT image acquisition was done by SIEMENS SOMATOM definition AS +128 and PHILLIP'S BRILLIANCE 64 SLICE at Kenyatta and Nairobi hospitals, respectively.

Nonenhanced scans were acquired first to demonstrate hemorrhage or calcification.

80 mls of LOCM (300mg/ml) via pump injector at injection rate of 5mls/second through an antecubital vein cannula (at least gauge 20) with a delay time of 7 seconds was used for acquisition of images in adult patients at Kenyatta National Hospital.

20 mls saline push before contrast administration, followed by 60 mls of LOCM at an injection rate of 4-5 mls/s via pump injector and 30 mls of saline chase with a scan delay time of 4.2 seconds, was used at Nairobi Hospital.

For young children and infants, a 22- or 24-gauge IV catheter was used and LOCM used at an injection rate of 2 mls/second.

A total of 94 patients (53 males and 41 females) referred for cerebral CTA at Kenyatta National Hospital and Nairobi Hospital from August 2017 to February 2018 were included. MIP and 3D cerebral CTA images were analyzed for anatomic variants and pathology. A cut-off value 0.8 mm vessel diameter was used. Any vessel less than 0.8 mm was considered hypoplastic/absent. Final configuration and pathology were confirmed by 2 senior radiologists. Patient's age, gender, anatomic variants of the circle of Willis, and presence of aneurysms and AVMs were recorded in the patient data sheet.

Statistical analysis using SPSS version 20.0 was done to determine association between the variants with aneurysms and arteriovenous malformations. P value was set at 0.05.

## 3. Results

Male patients accounted for 56.4% while 43.6% were female patients with and approx. male to female ratio of 1:1.

**Figure 1:**
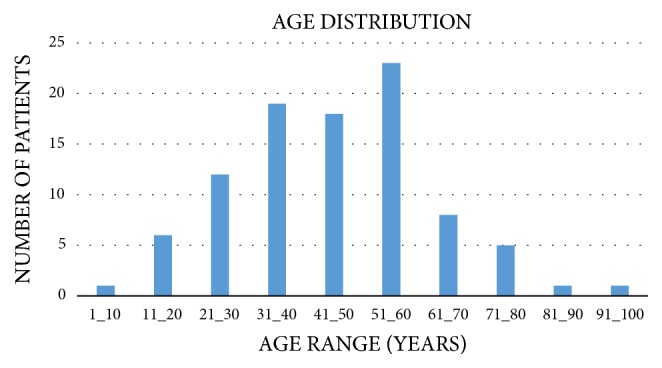
Age distribution of patients referred for cerebral CTA.

The age distribution of patients referred for cerebral CTA is depicted in Figure 1.

Majority of patients were referred for cerebral CTA due to clinically suspected intracranial hemorrhage accounting for 30.85%. Other clinical indications included suspected AVMs, aneurysms, cerebral sinus thrombosis, stroke, trauma, and tumor.

## 4. Proportion of Complete vs. Incomplete Circle of Willis

A complete CW was seen in 37.2% of the patients with no statistically significant difference between males and females (p=0.9) as shown in Table 1.

**Figure 2:**
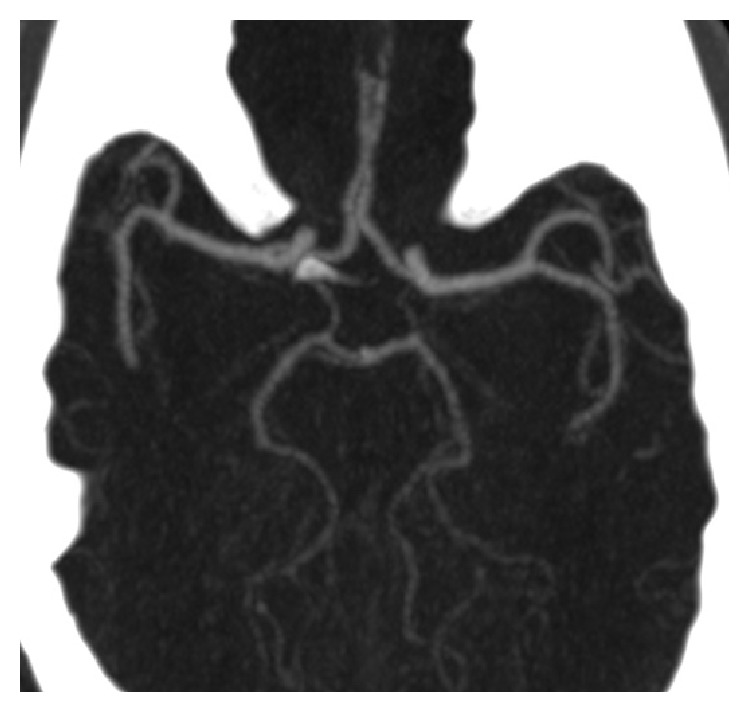
Axial cerebral CTA MIP image showing a complete CW configuration.

Figure 2 depicts a patient with complete circle of Willis in our study.

## 5. Anterior and Posterior Circulation Variants

Type A variant accounted for 78.7% of the anterior circulation variants and was significantly higher in females than male patients (p=0.016) as demonstrated in Table 2.

**Figure 3:**
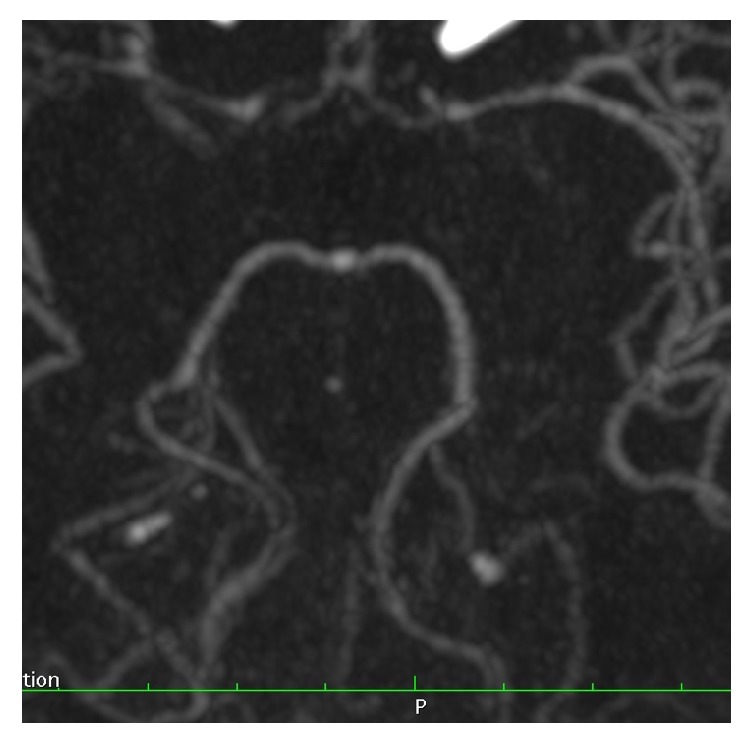
Axial MIP image showing combined type A anterior circulation variant and type E posterior circulation variant.

Figure 3 demonstrates a patient with combined type A anterior and type E posterior circulation variants.

Type E posterior circulation variant was the commonest seen accounting for 41.5% of the posterior circulation variants (Table 3).

## 6. Complete Anterior Circulation Variants (TYPES A-F)

A complete anterior CW configuration was seen in 84%. The proportion of this configuration was significantly higher in females than males (92.7% vs. 77.4%, p=0.044).

## 7. Complete Posterior Circulation Variants (TYPES A-C)

A complete posterior CW configuration was seen in 39.4% (39.6% vs. 39% in males and females, respectively, p=0.953).

### 7.1. Adult Configuration of the Posterior Cerebral Arteries

There was a significantly higher proportion of adult PCA configuration in females than males (95%vs 81% p=0.044).

## 8. Fetal PCA

**Figure 4:**
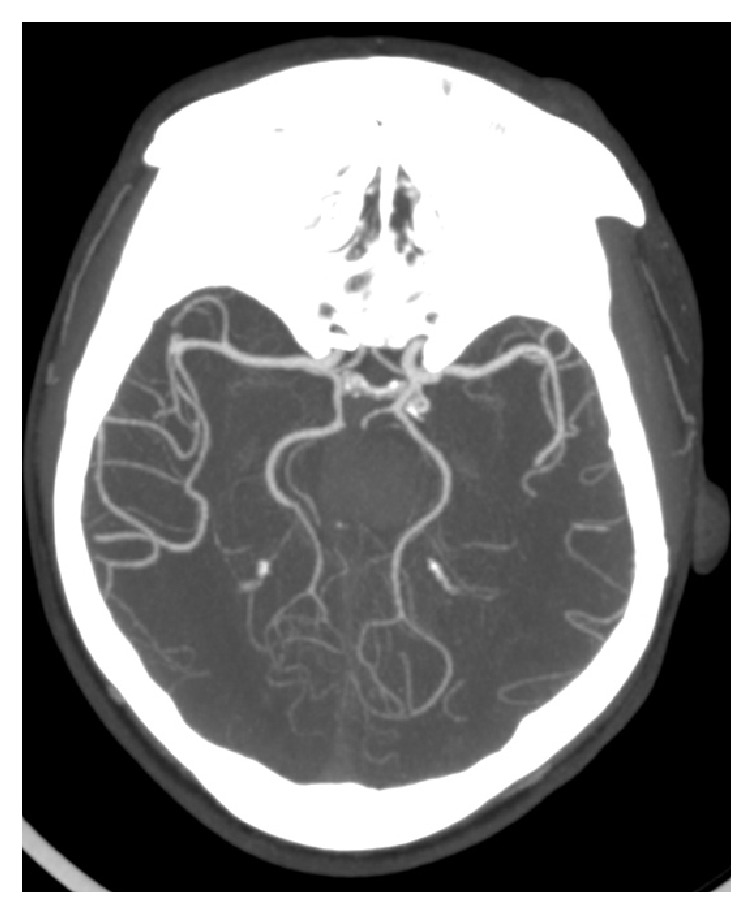
Axial cerebral CTA image showing bilateral fetal PCA with right P1 segment aplasia.

Fetal PCA (Figure 4) was seen in 25.5% (28.3% vs. 22.0% in males and females, respectively, p=0.484).

## 9. Presence of Aneurysms and Location

Aneurysms were seen in 24.5% of the patients referred for cerebral CTA (Table 4).

**Figure 5:**
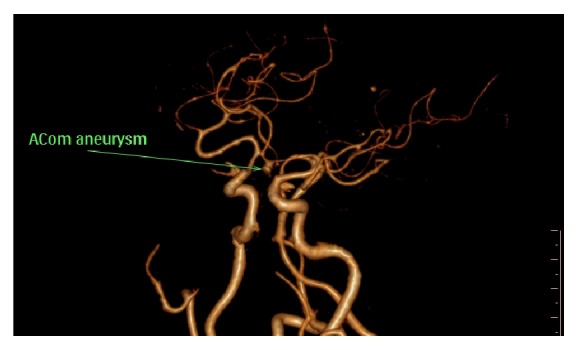
3D cerebral CTA showing a saccular aneurysm at the ACoA.

Figure 5 demonstrates a saccular aneurysm involving the ACoA.

## 10. Association of CW Configuration and Aneurysms

There was no association between CW configuration and presence of aneurysms as shown in Table 5.

## 11. Presence of Cerebral Arteriovenous Malformations

**Figure 6:**
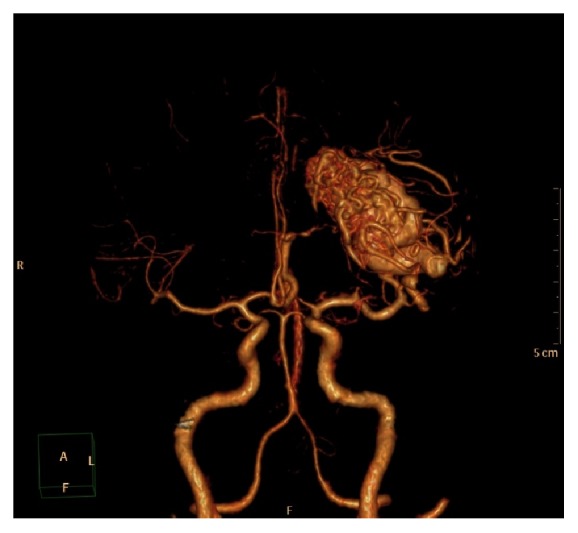
3D cerebral CTA image showing an AVM with feeding artery arising from the left MCA.

AVMs were seen in 8 (8.5%) of patients undergoing cerebral CTA (Figure 6). No association was found between CW configuration and presence of AVM (P=0.987).

Fenestrations, duplications, azygous ACA, and persistent TA were not observed in this study.

## 12. Discussion

Previous anatomic, CTA, and MRA studies have demonstrated significant variations in the configuration of the CW in various population groups.

The purpose of this study was to determine the proportion of variant anatomy of the circle of Willis (CW) and associated anomalies in patients with suspected cerebrovascular disease referred for cerebral CTA in 2 tertiary Kenyan hospitals. No similar studies on the CW configuration have been done in Kenya.

Variations in the CW configuration have been associated with cerebrovascular diseases [20, 21].

Various methods have been proposed for classification of variant anatomy of CW. The classification of CW configuration in this study is based on the Chen et al. classification which classified anterior and posterior variants into 10 groups (A-J) [18, 19].

The proportion of patients with complete CW configuration was 37.2% (n=35). This was slightly higher in males than females: 37.7% vs. 36.6%. The findings in the current study are consistent with previous studies which have demonstrated a prevalence of between 12.24%-60% [19, 22]. An MRA-TOF study by Chuanya Qiu et al. on 2246 healthy Chinese male population showed a lower prevalence of complete CW at 12.24% [4]. In contrast, an Egyptian MRA-TOF study by Mohammed Abdelatif et al. on 180 patients with no manifestations of cerebrovascular disease showed a higher prevalence of complete CW in 46.7% [23]. In our study, patients with cerebrovascular diseases referred for cerebral CTA were included unlike the above two MRA-TOF studies which were conducted on patients with no manifestations of cerebrovascular disease. The findings in the current study could thus be attributed to the difference in the study population, technique used, racial differences, and the lower sample size used in this study.

An incomplete CW configuration was seen in 62.8% of patients, 62.3% in males versus 63.4% in females. Previous studies have demonstrated a correlation between an incomplete CW and stroke severity and prognosis [10, 24].

According to the Chen et al., classification type A-F anterior variants are considered to be complete [18]. In our study, a complete anterior CW was seen in 84% (n=79). A complete anterior CW was significantly higher in female than male patients (92.7% vs. 77.4%, p=0.04). The prevalence of complete anterior CW in this study was higher than an MRA-TOF study by Sherif Mohamed Zaki et al. on 100 patents with no evidence of cerebrovascular disease which showed a prevalence of 66% [25]. The finding in our study was also higher than an MRA-TOF study by Shartri J et al. on 513 adults without cerebrovascular disease which demonstrated a complete anterior CW in 64.3% [26]. The higher prevalence of a complete anterior CW in our study could be due to higher spatial resolution of CTA as compared to MRA, sample size differences, and racial differences.

The commonest anterior circulation variant was type A variant accounting for 78.7% (n=74). This configuration was seen in 69.8% (n=37) and 90.2% (n=37) of males and females, respectively. This finding is similar to studies done by Naveen SR et al. and Dr. Arjun Bhaddur et al. which showed type A variant being the most common anterior circulation variant [27, 28].

Type E posterior variant has been associated with increased risk of ischaemic stroke [8]. This variant is of importance to surgeons and neurologists in the evaluation of patients with intracranial tumors, traumatic injuries, and cardiovascular complications [29].

In our study, type E posterior circulation variant was the commonest posterior circulation variant accounting for 41.5%. This study finding is similar to findings on an MRA-TOF study by Reddy Ravikanth et al. which demonstrated a predominance of type E posterior circulation at 53% [30].

A cerebral CTA study done by Zhang Ning Jin et al. on Chinese population with family history of stroke showed dominance of type E posterior variant with a prevalence of 52.3% [4], which is higher than the current study. The slight difference in prevalence of type E posterior variant could be due to racial and sample size differences.

In the current study, fetal PCA was seen in 30.9% (n=29). Complete PCA was demonstrated in 6.4% while partial PCA was seen in 24.5%. Unilateral fetal PCA was more common than bilateral fetal PCA. 12.8% were right sided, 10.6% left sided, and 5.6% bilateral fetal PCA.

These findings are similar to a publication by Dimmick SJ Faulder et al. which show a prevalence of 15%-32% for fetal PCA with bilateral fPCA at 8%, right sided at 10%, and left sided fPCA at 10% [13]. Fetal PCA has been found to increase the extent and severity of anterior circulation stroke as well as aneurysm formation [11, 31].

In the current study, aneurysms were seen in 24.5% (n=23) of patients referred for cerebral CTA. 69.6% of the aneurysms were located in the anterior CW while 4.3% were located in the posterior CW. 78.3% of patients had one aneurysm while 21.7% had more than 1 aneurysm.

26.1% (n=6) were located in other vessels other than the CW. The prevalence of aneurysms in this study is lower than that in an earlier Kenyan study by Chepsiror et al. based on conventional angiography which showed a higher prevalence of aneurysms at 40.9% and anterior circulation aneurysms at 95.2% [32]. The study by Chepsiror et al. was done on 88 patients with suspected cerebrovascular disease who were referred for conventional angiography in the two tertiary hospitals in Kenya. The difference in general prevalence of aneurysms in the two studies could be due to the higher sensitivity of conventional angiography for aneurysm detection.

In the current study, ACoA aneurysms were the commonest at 43.6% (n=10).

21.7% were located in the MCA, 21.7% were located in the ICA, and 13% were seen in other vessels than the CW. These findings are similar to a multimodality study on size and location of ruptured intracranial aneurysms by Young Gyun et al. which showed that a majority of aneurysms were located in the ACoA [33]. These study findings are, however, in contrast to a conventional angiographic study on pattern of cerebrovascular disease in Kenyatta and Nairobi hospitals done by Chepsiror et al. which showed 30.6% at ICA-PCoA, ACoA 22.2%, and MCA bifurcation 13% [32].

AVMs were seen in 8.5% (n=8) in the current study.

62.5% (n=5) had incomplete CW while 37.5% (n=3) had a complete CW configuration.

These study findings are similar to an earlier Kenyan conventional angiographic study by Chepsiror et al. which showed a prevalence of 8% [32].

Association between duplication and fenestration of intracranial arteries with aneurysmal formation has been documented in previous studies [13, 34]. Fenestration and duplication of CW were not observed in our study. Previous publication by Dimmick SJ Faulder et al. has shown a low prevalence for duplications and fenestrations in angiographic studies of 0.058% [13]. A similarly low prevalence of anterior circulation fenestration at 0.48% was demonstrated in a study by Roger M et al. on 411 patients undergoing cerebral CTA [35].

In the study by Roger M et al., patients with intracranial pathology such as subarachnoid hemorrhage, stroke, tumor, and aneurysms were excluded from the study. In a previous Kenyan cadaveric study by Hassan Said et al. on 36 adult brains, fenestration and duplication of ACoA were seen in 26% and 13%, respectively [36]. Another study by Dhanalakshmi V et al. on 50 adult cadaveric brains showed a relatively lower prevalence of duplications and fenestration of the ACoA at 8% and 4%, respectively [37]. The absence of fenestration and duplication in our study could be due to the general low incidence of duplications and fenestrations on angiographic studies, low sample size, and the unexplained discrepancy between angiographic and cadaveric studies.

Persistent trigeminal artery was not demonstrated in the current study. There is a general low prevalence of persistent trigeminal artery at 0.1-0.6% of all cerebral angiograms [38]. The absence of this variant could be explained based on the low prevalence in the general population and the low sample size used in this study.

In our study, no case of azygous anterior cerebral artery was identified. In a previous publication on aneurysms of the azygous anterior cerebral artery by Rajesh Kumar et al., prevalence of azygous ACA was quoted at <1% with a range of 0.21%-3.73% [39]. The finding in our study could be attributed to the low prevalence of azygous ACA in the general population and the lower sample size.

In the current study, no association was found between the CW configuration and presence of aneurysms (p=0.567). These findings are similar to a retrospective cerebral CTA study by P. Brzegowy et al. which showed no association between anatomic variations of the MCA and CW with aneurysms [40].

There was no significant correlation between CW configuration and occurrence of AVMs (P=0.987).

## 13. Conclusion

The findings in this study demonstrate slight differences in the CW configuration. A higher proportion of complete anterior CW was seen in female patients. Type A anterior and type E posterior circulation variants were the commonest in both males and females. No significant association was demonstrated between CW configuration and occurrence of aneurysms/AVMs in this study. Various methods of classification of CW configuration have been proposed with no standardized method to date, thus the need for consensus building between neuroanatomists, neurologists, neurosurgeons, and neuroradiologists for ease of comparison between similar future studies on the CW.

## Figures and Tables

**Table 1:** Percentage distribution of complete vs incomplete CW in males vs female patients.

GENDER	PERCENTAGE DISTRIBUTION OF CW CONFIGURATION	
	COMPLETE	INCOMPLETE
MALE	37.7% (*n* = 20)	62.3% (*n* = 33)
FEMALE	36.6% (*n* = 15)	63.4% (*n* = 26)
COMBINED(MALE AND FEMALE)	37.2% (*n* = 35)	62.8% (*n* = 59)

**Table 2:** Anterior circulation variant percentage distribution.

ANTERIOR CW	FREQUENCY (IN BOTH MALE AND FEMALES)	FREQUENCY IN MALES	FREQUENCY IN FEMALES
TYPEA	78.7% (n=74)	69.8% (n=37)	90.2% (n=37)
TYPE B	0% (n=0)		
TYPE C	1.1% (n=1)		
TYPE D	4.3% (n=4)		
TYPE E	0% (n=0)		
TYPE F	0% (n=0)		
TYPE G	4.3% (n=4)		
TYPE H	11.7% (n=11)		
TYPE I	0% (n=0)		
TYPE J	0% (n=0)		

**Table 3:** Proportion of posterior circulation variants.

POSTERIOR CW	FREQUENCY
TYPE A	27.7% (26)
TYPE B	4.3% (4)
TYPE C	7.4% (7)
TYPE D	4.3% (4)
TYPE E	41.5% (39)
TYPE F	2.1% (2)
TYPE G	7.4% (7)
TYPE H	0% (0)
TYPE I	0% (0)
TYPE J	5.3% (5)

**Table 4:** Distribution of intracranial aneurysms.

Location of aneurysms		
ACOA	10	43.60%
MCA	5	21.70%
ICA	5	21.70%
Other vessels than	3	13.00%

**Table 5:** Presence and number of aneurysms in complete and incomplete CW.

	Total	Circle of WILLS anatomy		P value
Characteristics		Incomplete	Complete	
*Presence of Aneurysm*				
No	71(75.5%)	43(72.9%)	28(80.0%)	0.438
Yes	23(24.5%)	16(27.1%)	7(20.0%)	

*Number of Aneurysms*				
1 Aneurysm	18(78.3%)	12(75.0%)	6(85.7%)	0.567
More than 1 Aneurysm	5(21.7%)	4(25.0%)	1(14.3%)	

## Data Availability

Original data can be accessed upon request.

## References

[1] Kathuria S., Gregg L., Chen J., Gandhi D. (2011). Normal cerebral arterial development and variations. *Seminars in Ultrasound, CT and MRI*.

[2] Rosner J., Lui F. (2019). Neuroanatomy, Circle of Willis. *StatPearls*.

[3] Takakuwa T., Koike T., Muranaka T., Uwabe C., Yamada S. (2016). Formation of the circle of Willis during human embryonic development. *Congenital Anomalies*.

[4] Qiu C., Zhang Y., Xue C., Jiang S., Zhang W. (2015). MRA study on variation of the circle of Willis in healthy Chinese male adults. *BioMed Research International*.

[5] Carlson B. M. (2014). *Human Embryology and Developmental Biology*.

[6] Menshawi K., Mohr J. P., Gutierrez J. (2015). A functional perspective on the embryology and anatomy of the cerebral blood supply. *Journal of Stroke*.

[7] Lazzaro M. A., Ouyang B., Chen M. (2011). The role of circle of Willis anomalies in cerebral aneurysm rupture. *Journal of NeuroInterventional Surgery*.

[8] van Seeters T., Hendrikse J., Biessels G. J. (2015). Completeness of the circle of Willis and risk of ischemic stroke in patients without cerebrovascular disease. *Neuroradiology*.

[9] Polguj M., Majos M., Topol M., Majos A. (2014). An asymmetrical fenestration of the basilar artery coexisting with two aneurysms in a patient with Subarachnoid haemorrhage: Case report and review of the literature. *Folia Morphologica (Poland)*.

[10] Zhou H., Sun J., Ji X. (2016). Correlation between the integrity of the circle of willis and the severity of initial noncardiac cerebral infarction and clinical prognosis. *Medicine*.

[11] He Z., Wan Y. (2018). Is fetal-type posterior cerebral artery a risk factor for intracranial aneurysm as analyzed by multislice CT angiography?. *Experimental and Therapeutic Medicine*.

[12] Chuang Y.-M., Chan L., Lai Y.-J. (2013). Configuration of the Circle of Willis is associated with less symptomatic intracerebral hemorrhage in ischemic stroke patients treated with intravenous thrombolysis. *Journal of Critical Care*.

[13] Dimmick S. J., Faulder K. C. (2009). Normal variants of the cerebral circulation at multidetector CT angiography. *RadioGraphics*.

[14] Yang Z. L., Ni Q. Q., Schoepf U. J. (2017). Small intracranial aneurysms: Diagnostic accuracy of CT angiography. *Radiology*.

[15] Dorcas H. P. (1948). The development of the cranial arteries in the human embryo. *Contributions to Embryology*.

[16] Zurada A., Gielecki J. S. (2007). A novel formula for the classification of blood vessels according to symmetry, asymmetry and hypoplasia. *Folia Morphologica*.

[17] Krabbe-Hartkamp M. J., Van Der Grond J., De Leeuw F.-E. (1998). Circle of Willis: morphologic variation on three-dimensional time-of- flight MR angiograms. *Radiology*.

[18] Li Q., Li J., Lv F., Li K., Luo T., Xie P. (2011). A multidetector CT angiography study of variations in the circle of Willis in a Chinese population. *Journal of Clinical Neuroscience*.

[19] Chen H.-W., Yen P.-S., Lee C.-C. (2004). Magnetic resonance angiographic evaluation of circle of Willis in general population: a morphologic study in 507 cases. *Chinese Journal of Radiology-Taipei*.

[20] Ogeng’o J. A., Mwachaka P. M., Odhiambo D. E. (2018). Geometric features of vertebrobasilar arterial system in adult black kenyans. *International Journal of Morphology*.

[21] Liu Z., Cai Y., Chen G.-Z., Lu G.-M., Li Z.-Y. (2017). Anatomical variations in circle of willis and intracranial aneurysm formation. *Molecular & Cellular Biomechanics*.

[22] Krabbe-Hartkamp M. J., Van Der Grond J., Van Everdingen K. J., Hillen B., Mali W. P. T. M. (1999). Circle of Willis collateral flow investigated by magnetic resonance angiography. *Stroke*.

[23] Maaly M. A., Ismail A. A. (2011). Three dimensional magnetic resonance angiography of the circle of Willis: anatomical variations in general Egyptian population. *The Egyptian Journal of Radiology & Nuclear Medicine*.

[24] Zhou C., Yuan C., Li R., Wang W., Li C., Zhao X. (2018). Association between incomplete circle of willis and carotid vulnerable atherosclerotic plaques. *Arteriosclerosis, Thrombosis, and Vascular Biology*.

[25] Zaki S. M., Shaaban M. H., Abd Al Galeel W. A., El Husseiny A. A. (2019). Configuration of the circle of Willis and its two parts among Egyptian: a magnetic resonance angiographic study. *Folia Morphologica*.

[26] Shatri J., Cerkezi S., Ademi V., Reci V., Bexheti S. (2018). Anatomical variations and dimensions of arteries in the anterior part of the circle of Willis. *Folia Morphologica*.

[27] Naveen S. R., Bhat V., Karthik G. A. (2015). Magnetic resonance angiographic evaluation of circle of Willis: A morphologic study in a tertiary hospital set up. *Annals of Indian Academy of Neurology*.

[28] Arjun Bahaddur, Chandan G. (2013). Anatomic variants of circle of willis in South Indian population:a study by using magnetic resonance angiography. *International Journal of Science Research*.

[29] Sussman E. S., Kellner C. P., Mergeche J. L. (2014). Radiographic absence of the posterior communicating arteries and the prediction of cognitive dysfunction after carotid endarterectomy: Clinical article. *Journal of Neurosurgery*.

[30] Ravikanth R., Philip B. (2019). Magnetic resonance angiography determined variations in the circle of Willis: Analysis of a large series from a single center. *Tzu Chi Medical Journal*.

[31] Lambert S. L., Williams F. J., Oganisyan Z. Z., Branch L. A., Mader E. C. (2016). Fetal-type variants of the posterior cerebral artery and concurrent infarction in the major arterial territories of the cerebral hemisphere. *Journal of Investigative Medicine High Impact Case Reports*.

[32] Chepsiror D. K. (2006). *The pattern of radiological findings seen in four-vessel cerebral angiography done at the KNH and Nairobi Hospital [Thesis]*.

[33] Jeong Y.-G., Jung Y.-T., Kim M.-S., Eun C.-K., Jang S.-H. (2009). Size and location of ruptured intracranial aneurysms. *Journal of Korean Neurosurgical Society*.

[34] Sanders W. P., Sorek P. A., Mehta B. A. (1993). Fenestration of intracranial arteries with special attention to associated aneurysms and other anomalies. *American Journal of Neuroradiology*.

[35] Krzyżewski R. M., Tomaszewski K. A., Kochana M., Kopeć M., Klimek-Piotrowska W., Walocha J. A. (2014). Anatomical variations of the anterior communicating artery complex: gender relationship. *Surgical and Radiologic Anatomy*.

[36] Hassan Profs S. H., Kitunguu P., Ogengo J. A. (2008). Variant anatomy of the anterior cerebral artery in Adult Kenyans. *African Journal of Neurological Sciences*.

[37] Lakshmi D., Kumar S., Kumar S. T., kumar K. A. (2019). Anterior communicating artery: a cadaveric study. *International Journal of Anatomy and Research*.

[38] Tomalczyk A., Kaurzel Z., Szubert W., Oszukowski P. (2017). Arteria trigemina primitiva - radiological report of three cases and review of literature. *Folia Morphologica (Poland)*.

[39] Ghanta R., Kesanakurthy M., Vemuri V. (2016). Aneurysm of azygos anterior cerebral artery: A report of two cases. *Asian Journal of Neurosurgery*.

[40] Brzegowy P., Polak J., Wnuk J., Łasocha B., Walocha J., Popiela T. J. (2018). Middle cerebral artery anatomical variations and aneurysms: A retrospective study based on computed tomography angiography findings. *Folia Morphologica (Poland)*.

